# The dose-dependent anesthetic effect of ciprofol on 8-week-old C57BL/6J mice

**DOI:** 10.3389/fphar.2026.1842059

**Published:** 2026-05-28

**Authors:** Huan Wang, Yongliang Chi, Zhaobing Han, Xiwen Geng, Guimao Cao

**Affiliations:** 1 The Second Clinical Medical College, Shandong University of Traditional Chinese Medicine, Jinan, China; 2 Department of Anesthesiology, Affiliated Hospital of Shandong University of Traditional Chinese Medicine, Jinan, China; 3 Experimental Center, Shandong University of Traditional Chinese Medicine, Jinan, China

**Keywords:** 95% effective dose (ED_95_), C57BL/6J mice, ciprofol, half effective dose (ED_50_), righting reflex

## Abstract

**Objective:**

The research is to explore the half effective dose (ED_50_) and 95% effective dose (ED_95_) of ciprofol with intraperitoneal injection of 8-week-old C57BL/6J adult mice, which will provide experimental basis for the reasonable dose design of ciprofol in preclinical basic research.

**Methods:**

C57BL/6J mice were selected and divided into 8 dose groups (50, 55, 60, 65, 70, 80, 90, 100 mg/kg), with 12 males and 12 females in each group. The ciprofol was administered by intraperitoneal injection, and the latency of loss of righting reflex (LORR) and recovery time of righting reflex (RORR) were recorded. Probit regression analysis was used to calculate the ED_50_ and ED_95_ values of ciprofol anesthesia in mice.

**Results:**

There was a significant positive correlation between the dose of ciprofol and the positive rate of anesthesia in mice, and the recovery time of anesthesia was prolonged with the increase of dose. The ED_50_ of ciprofol in anesthesia was 56.014 mg/kg for male and 54.783 mg/kg for female, and the ED95 was 91.622 mg/kg for male and 82.212 mg/kg for female. There was no significant difference in the anesthetic dose between male and female mice.

**Conclusion:**

In this study, the ED_50_ and ED_95_ of ciprofol in male and female mice was determined. Our results demonstrate that ciprofol induces a dose-dependent anesthetic effect in mice, but gender has no significant impact on its efficacy. Collectively, these data provide a critical dosimetric basis for the standardized application of ciprofol in preclinical research.

## 1 Introduction

As the first type 1 intravenous anesthesia innovative drug independently developed in China (Lu et al., 2023), ciprofol is a new type of 2,6-disubstituted phenol derivative (Qin et al., 2017). It is the chiral structure optimization product of the classical intravenous anesthetic propofol. Its chemical structure is (R) -2- (1-cyclopropylethyl) -6-isopropylphenol (Lu et al., 2023). γ-aminobutyric acid type A (GABA_A_) receptors exhibit stereoselectivity for anesthetic agents (Lingamaneni and Hemmings, 2003). By introducing a cyclopropyl group into the propofol molecular structure to form a chiral center, the drug enhances stereospecific interaction, thereby increasing its binding affinity for GABA_A_ receptors to 4–5 times that of propofol (Wei et al., 2017; Liao et al., 2022). Compared with propofol, ciprofol has higher clinical application safety, which can effectively reduce the incidence of adverse reactions such as injection pain (Wang et al., 2022), respiratory depression, and cardiovascular function fluctuations. At the same time, it has the pharmacological characteristics of fast metabolism, rapid and thorough recovery (Wei et al., 2017), which is in line with the core requirements of ' precise anesthesia and rapid recovery ' in clinical anesthesia. Since its listing, ciprofol has been widely used in many clinical scenarios such as general anesthesia induction and maintenance, endoscopic sedation, and sedation of critically ill patients, and has become one of the main drugs for clinical intravenous anesthesia. With the continuous expansion of clinical application scenarios of propofol, the demand for basic experimental research on its pharmacological mechanism, prevention and control of adverse reactions, and optimization of combination regimens is increasing.

Mouse is the most commonly used model organism in pharmacological basic research, and its standardized anesthetic dosage is the core basic parameter for the study of pharmacodynamics, toxicology, pharmacokinetics and receptor binding mechanism of ciprofol. At present, there is no clear literature report on the standardized anesthetic dose of ciprofol in mice at home and abroad. This research gap seriously limits the development of relevant basic experiments and the clinical transformation of research results. Based on this, in this study, 8-week-old C57BL/6J mice were used as experimental subjects, and different doses of ciprofol were injected intraperitoneally to explore the effective dose of anesthesia. The purpose of this study is to provide a reliable dosimetric basis for the basic experimental research of ciprofol and promote the standardization and scientification of the basic research of ciprofol.

## 2 Materials and methods

### 2.1 Experimental animals

In this study, SPF-grade C57 mice aged 8 weeks, with equal numbers of males and females (weight 20–25 g), were purchased from Jiangsu Huachuang Xinnuo Pharmaceutical Technology Co., Ltd. animal production license number: SCXK (Su) 2020-0009, user license number: SYXK (Lu) 20220009. The mice were raised in a barrier environment with a temperature of (23 ± 2) °C and a humidity of (50 ± 5) %. The light and dark cycles were alternated for 12 h, and the mice were fed with standard feed and drinking water freely. The experimental process strictly followed the ‘Laboratory Animal Ethics Review Guideline (GB/T 35892-2018)’ and was approved by the Animal Ethics Review Committee of Shandong University of Traditional Chinese Medicine (approval No.: SDUTCM20250414001).

### 2.2 Experimental method

In this study, eight dose gradients of 50, 55, 60, 65, 70, 80, 90 and 100 mg/kg were set up, with 12 male and 12 female mice in each group. Ciprofol (brand name: Sishuning, batch No.33251016) was injected intraperitoneally as a single bolus injection without solvent dilution. Each dose was calculated based on individual body weight (mg/kg) on the day of the experiment. The injection was administered using a 1 mL syringe with a 26 gauge needle at a constant rate of approximately 0.1 mL/s to ensure consistency across all animals. The disappearance of righting reflex was used as the core csissrion for the onset of anesthesia. After intraperitoneal injection, the mice were placed in a cage alone for continuous observation. If the mice could not recover the prone position within 10 s after being placed in the supine position (Wada et al., 2024), it was judged as the disappearance of righting reflex, which was recorded as the positive reaction of anesthesia onset. During the experiment, the anesthetic indicators were recorded synchronously: latency of loss of righting reflex (LORR) (the time from drug administration to the disappearance of the righting reflex) and recovery time of righting reflex (RORR) (the time from the disappearance of the righting reflex to complete recovery). The anesthetic effect and maintenance effect of ciprofol were evaluated objectively and quantitatively based on these indicators.

### 2.3 Statistics and analysis

The anesthesia positive reaction rate of mice in each dose group was calculated, and the calculation formula was: anesthesia positive reaction rate = (number of anesthesia positive mice/total number of mice in this group) × 100%. SPSS 27.0 statistical software was used for data processing. Probit regression analysis was used to calculate the ED_50_, ED_95_ and 95% confidence interval (95% CI) of ciprofol for anesthesia in mice. The Wald test was used to compare the positive anesthesia response rates of male and female mice at the same dose, and to determine the gender difference, with P < 0.05 considered statistically significant. Simple linear regression was performed to assess the associations between ciprofol dose and anesthetic indicators, including LORR latency and RORR time, in male and female mice separately. Where applicable, comparisons of regression slopes and intercepts between sexes were conducted to detect sex-related differences. The threshold for statistical significance was defined as P < 0.05 (α = 0.05). Measurement data were expressed as mean ± standard deviation (x̄ ± s). For probit regression, model fit was assessed using the Pearson goodness-of-fit test, with P > 0.05 indicating adequate fit. Standardized residuals were examined to identify potential outliers. For simple linear regression, the assumptions of normality of residuals (Shapiro-Wilk test) and independence of errors were verified. The coefficient of determination (R^2^) was calculated for each regression model.

## 3 Results

### 3.1 Dose-effect relationship and ED_50_/ED_95_ calculation of ciprofol for anesthesia in mice

The dose of ciprofol was positively correlated with the positive reaction rate of anesthesia in mice in a dose-dependent manner. With the increase of the dose from 50 mg/kg to 100 mg/kg, the positive reaction rate of anesthesia in mice showed a continuous upward trend. In the 50 mg/kg dose group, the positive reaction rate of male mice was 42%, and that of female mice was 50%. With the increase of dosage, the positive reaction rate gradually increased. When the dosage reached 90 mg/kg, the positive reaction rate of anesthesia in both male and female mice reached 100%, and the positive reaction rate in the 100 mg/kg dose group maintained 100%. The results of Probit regression analysis showed that there was a significant linear correlation between the dose of ciprofol and the positive reaction rate of anesthesia in mice (P < 0.001).

**FIGURE 1:**
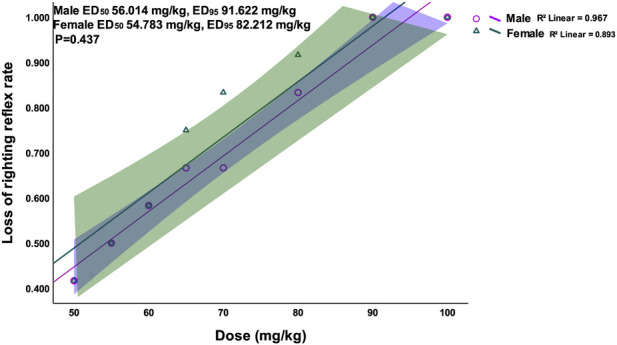
Dose-effect relationship curve of ciprofol anesthesia in C57BL/6J mice. After intraperitoneal injection of ciprofol, the positive reaction rate of anesthesia in male and female C57BL/6J mice increased in a dose-dependent manner with the increase of administration dose. The ED50 of male mice was 56.014 mg/kg, and the ED95 was 91.622 mg/kg. The ED50 of female mice was 54.783 mg/kg, and the ED95 was 82.212 mg/kg.

Probit regression analysis showed that the ED_50_ of male C57BL/6J mice was 56.014 mg/kg, and the 95% CI was 47.536–61.257 mg/kg. The ED_95_ of anesthesia was 91.622 mg/kg (95% CI: 79.828–129.661 mg/kg). The ED_50_ of ciprofol for female C57BL/6J mice was 54.783 mg/kg, and the 95% CI was 47.403–59.304 mg/kg. The ED_95_ of anesthesia was 82.212 mg/kg (95% CI: 73.509–107.604 mg/kg) (Figure 1). Model diagnostics for probit regression revealed adequate goodness-of-fit for both male and female datasets (Pearson Goodness-of-Fit Test: male χ^2^ = 1.668, df = 6, P = 0.948; female χ^2^ = 0.511, df = 6, P = 0.998). No significant outliers were detected based on standardized residuals.

### 3.2 Effects of gender factors on the anesthetic dose of ciprofol in mice

The positive reaction rate of anesthesia in male and female mice in each dose group was statistically compared. The results showed that there was no significant difference in the positive reaction rate of anesthesia between male and female mice in all dose groups (P = 0.437), suggesting that gender factors had no significant effect on the effective dose of anesthesia in C57BL/6J mice.

### 3.3 Effects of ciprofol on LORR latency and RORR time in mice

**FIGURE 2:**
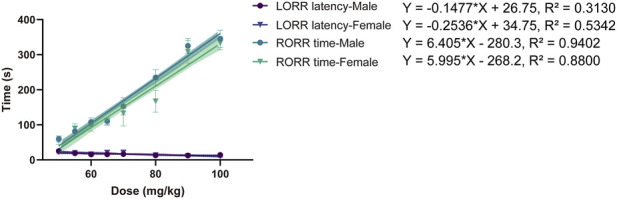
Linear correlation between ciprofol dose and LORR latency/RORR time in male and female mice. Data are presented as mean ± standard deviation (SD). Solid lines represent the best-fit linear regression for each group. LORR latency was negatively correlated with dose, while RORR time was positively correlated with dose in both sexes (p < 0.0001). For *R* ^2^ values and model diagnostics, see Results section.

With increasing doses of ciprofol, the LORR latency in mice showed a significant decreasing trend, while the RORR time was significantly prolonged. Compared with the 50 mg/kg group, all higher doses produced statistically significant differences in both parameters in male mice (P < 0.001). In female mice, while most doses exhibited significant differences, two intermediate doses did not reach statistical significance relative to the 50 mg/kg baseline (P > 0.05), yet the overall dose-dependent pattern remained consistent. (Figure 2). Linear regression analyses showed that dose significantly predicted both LORR latency and RORR time in both sexes. For LORR latency, the proportion of variance explained by dose (*R* ^2^) was 0.3130 in males and 0.5342 in females; residual analysis was 3.664 in males and 3.964 and 26.40 and 36.17 in females; regression equations was Y = −0.1477*X + 26.75 in males and Y = −0.2536*X + 34.75 in females. For RORR time, *R* ^2^ was 0.9402 in males and 0.8800 in females; residual analysis was 26.40 in males and 36.17 in females; regression equations was Y = 6.405*X −280.3 in males and Y = 5.995*X - 268.2 in females. Residual analysis confirmed normality (Shapiro-Wilk P > 0.05 for all models) and independence of residuals was assessed via visual inspection of residual plots, which showed no evidence of systematic trends or serial correlation. No clustering or periodic patterns were observed. The specific data of righting reflex disappearance and recovery time of male and female mice in each dose group are shown in Table 1.

**TABLE 1:** Effects of different doses of ciprofol on the disappearance and recovery time of righting reflex in C57BL/6J mice (xˉ ± s, min).

Dose (mg/kg)	LORR latency (min)		RORR time (min)	
	Male	Female	Male	Female
50	25.20 ± 3.77	21.80 ± 5.59	59.60 ± 8.80	55.60 ± 12.12
55	18.83 ± 1.47^②^	19.83 ± 2.32	81.50 ± 14.63	89.50 ± 13.56
60	15.57 ± 2.88^④^	15.86 ± 2.27^①^	108.00 ± 11.94^③^	95.71 ± 14.19
65	15.75 ± 3.37^④^	20.67 ± 5.77	110.13 ± 9.73^③^	108.89 ± 10.33^②^
70	16.63 ± 1.20^④^	21.10 ± 4.68	152.88 ± 24.25^④^	133.20 ± 36.24^④^
80	12.90 ± 3.76^④^	13.00 ± 1.34^③^	235.20 ± 22.22^④^	171.09 ± 29.25^④^
90	12.50 ± 2.94^④^	11.17 ± 2.44^④^	325.33 ± 21.10^④^	308.50 ± 32.39^④^
100	14.25 ± 3.28^④^	9.42 ± 2.27^④^	345.25 ± 24.74^④^	333.00 ± 19.39^④^

Compared with the same group at 50 mg/kg, ① P < 0.05, ② P < 0.01, ③ P < 0.001, ④ P < 0.0001.

## 4 Discussion

In this study, based on the classical anesthetic evaluation model of loss of righting reflex in mice, the anesthetic dose-effect relationship of ciprofol in 8-week-old C57BL/6J mice was systematically determined for the first time when intraperitoneal injection was administered. The corresponding ED_50_ and ED_95_ of male and female mice were determined. At the same time, it was confirmed that the incubation period and recovery time of anesthesia showed typical dose-dependent characteristics, which provided quantitative data support for ciprofol in preclinical pharmacological research of rodents. Compared with the existing similar studies, the dose-effect parameters measured in this study fill the gaps in the pharmacodynamic data of ciprofol in the intraperitoneal injection mode of this strain of mice, and can provide a dose selection basis for the basic experiments such as anesthesia mechanism and combined medication carried out in subsequent relying mice.

From the perspective of anesthetic potency, the ED_50_ of ciprofol-induced anesthesia in mice was much lower than that of propofol (140 mg/kg) reported in the literature (Irifune et al., 1999), suggesting that ciprofol had better anesthetic activity. This result is highly consistent with the optimization of pharmacological characteristics brought by structural modification. As a structural derivative of propofol, ciprofol group is introduced into the core structure of the parent nucleus (Wei et al., 2017), which can significantly enhance the binding affinity of the drug with GABA_A_ receptor (Liao et al., 2022). GABA_A_ receptor is the core target of general anesthetics, and the increase of affinity directly promotes the equivalent anesthetic effect of ciprofol at a lower dose, which is also the key molecular mechanism of its anesthetic potency superior to propofol.

The results of this study showed that there was no significant difference in ED_50_ and ED_95_ between male and female mice, indicating that in the C57BL/6J strain, the interference of gender factors on the anesthetic effect of ciprofol was not significant, which simplified the design process and reduced the experimental deviation for the subsequent mixed sex animal experiments. In addition, the correlation between the dose of ciprofol and the time of anesthesia was observed in this study: the latency of loss of righting reflex was gradually shortened with the increase of dose, and the recovery time of righting reflex was gradually prolonged with the increase of dose. The dose-dependent characteristics were consistent with the efficacy of ciprofol in clinical application, which proved that the mouse model could better simulate the pharmacodynamic response of human body to ciprofol, and there was only a difference in dose requirements between species, which further proved the reference value of this study data for preclinical research. Compared with the administration characteristics of short-acting and fast metabolism, the characteristics of prolonged anesthesia recovery time of righting reflex at high doses suggest that the corresponding dose can be flexibly selected according to the requirements of anesthesia maintenance time in the experimental design, taking into account the stability of anesthesia and experimental efficiency.

Although this study has clarified the core anesthetic dose-effect relationship of ciprofol in C57BL/6J mice, there are still some limitations that need to be improved in subsequent studies.

First of all, the study only selected C57BL/6J single strain mice, not included in Kunming mice and other commonly used experimental strains. The genetic background and metabolic enzyme expression of different strains of mice are different, and there may be differences in the anesthetic sensitivity of ciprofol. The universality of the research conclusion needs to be further verified.

Secondly, This study is limited by the exclusive use of pharmacodynamic (PD) assessments without concurrent plasma ciprofol concentration measurements in mice, precluding a dose–concentration–effect tripartite correlation. Nonetheless, the observed dose–effect relationships are mechanistically interpretable based on ciprofol’s established pharmacokinetic (PK) and PD profiles. The intraperitoneal ED_50_ of ciprofol in mice was approximately 55–56 mg/kg, 2.5-fold lower than that of propofol (140 mg/kg) under identical conditions, indicating superior anesthetic potency attributable to its molecular-level pharmacological advantages. Although mouse-specific PK data were not obtained, PK data from other species adequately explain the observed anesthetic features. Ciprofol exhibits rapid and extensive tissue distribution, with a steady-state volume of distribution (V_ss_) of 7.79 L/kg in rats (Liao et al., 2022) —far exceeding total body water—indicating efficient penetration into peripheral tissues and the brain. This property supports rapid delivery to the central nervous system, consistent with dose-dependent loss of righting reflex (LORR) latencies (10–25 min). In humans (Bian et al., 2021), ciprofol follows tri-exponential elimination, with distribution half-lives of 2.0 min (α) and 34.9 min (β), and a terminal elimination half-life of 6.2 h. Despite interspecies quantitative differences, the conserved PK profile of rapid distribution followed by slow elimination underpins its anesthetic dynamics. The β-phase half-life, reflecting peripheral redistribution, primarily governs recovery of righting reflex (RORR). As coprophil redistributes from the brain to peripheral tissues, effect-site concentration declines below the hypnotic threshold. Higher doses increase systemic burden, prolonging the time to subthreshold effect-site concentration. This mechanism validates our findings: increasing the dose from 50 mg/kg to 90–100 mg/kg markedly prolonged RORR time from ∼55 min to >300 min, while shortening LORR latency from 20–25 min to 10–15 min. Metabolically, carotol is inactivated via rapid hepatic glucuronidation (UGT enzymes) and CYP2B6, producing inactive M4 glucuronide conjugates (Zhou et al., 2025). The absence of active metabolites and high metabolic clearance avoid cumulative sedation, accounting for the lack of mortality or persistent adverse effects even at 100 mg/kg in mice, with full recovery of righting reflex. Based on these PK/metabolic characteristics, a mechanistic framework is proposed: After intraperitoneal injection, ciprofol enters systemic circulation and rapidly distributes to the brain due to high lipophilicity and large V_ss_, achieving peak effect-site concentration within 10–15 min. Hypnosis onset (LORR) occurs when brain concentration exceeds the hypnotic threshold; higher doses accelerate the rise and increase peak concentration, shortening LORR latency. Anesthetic depth and duration are determined by the time effect-site concentration remains above threshold. Recovery begins upon redistribution and metabolism reducing brain concentration below threshold. Given the lack of active metabolites, RORR time depends primarily on effect-site elimination, prolonged dose-dependently by increased drug burden. Future studies should adopt classical PK–PD models (e.g., the effect-compartment-linked inhibitory sigmoidal E_max_ model, validated for correlating ciprofol plasma concentration with BIS values in humans) to quantify dose–effect relationships. Calibration of a mouse model—estimating effect-site equilibration rate constant (k_e0_), righting reflex-based EC_50_, and Hill coefficient—would enable prediction of anesthetic onset and duration and guide dose selection. The ED_50_ and ED_95_ values determined here provide a foundation for such modeling. Several limitations are acknowledged: (1) No published mouse-specific PK parameters for ciprofol exist; interspecies differences in metabolism, protein binding, and tissue distribution may cause quantitative bias in cross-species extrapolation. (2) Existing PK data derive from intravenous administration, whereas this study used intraperitoneal injection; the slower, more variable absorption of the intraperitoneal route may alter the correlation between administered dose and peak effect-site concentration. (3) Direct measurements of plasma and brain ciprofol concentrations in mice were not performed, so the proposed mechanistic framework remains hypothetical. Despite these limitations, the high consistency between our PD findings and the established PK, receptor-binding, and metabolic properties of ciprofol affirms the internal validity of this study. The dose-dependent LORR and RORR trends align with ciprofol’s PK characteristics, and its greater anesthetic potency relative to propofol is consistent with its 4–5-fold higher affinity for GABAA receptors. Collectively, these findings provide a rigorous, mechanism-based dosimetric basis for preclinical application of ciprofol. Despite these limitations, the consistency between our PD observations and the known PK, receptor-binding, and metabolic characteristics of ciprofol supports the internal validity of our findings. The dose-dependent changes in LORR latency and RORR time are coherent with the expected pharmacokinetic behavior of ciprofol, and the potency difference relative to propofol aligns with its 4 – 5 fold higher GABA_A_ receptor affinity. Together, these considerations provide a quantitative and mechanism-based dosimetric foundation for the rational use of ciprofol in preclinical research.

Third, the anesthesia evaluation index is limited to the sign of righting reflex disappearance, and the key physiological indexes such as cardiovascular and respiratory function of mice are not systematically monitored. While righting reflex is a well-established behavioral endpoint for hypnosis in rodent anesthesia research, it does not capture clinically relevant physiological domains such as cardiovascular function, respiratory drive, or nociceptive response. Ciprofol, like other GABA_A_ receptor agonists, can produce dose-dependent respiratory depression, hypotension, and bradycardia in clinical settings (Wang et al., 2022; Lu et al., 2023). Because we did not monitor oxygen saturation, respiratory rate, heart rate, or blood pressure in the present study, we cannot infer the safety margin of the tested doses from a cardiorespiratory perspective. Consequently, the ED_50_ and ED_95_values reported here should be interpreted as doses required to achieve hypnosis (loss of righting reflex) rather than doses that are necessarily safe or optimal for survival surgery. The absence of physiological monitoring also limits the translational validity of our findings: in humans, anesthetic depth is routinely guided by vital signs and electroencephalography, not by motor reflexes alone. Future studies should incorporate real-time monitoring of pulse oximetry, capnography, or electrocardiography to establish a more comprehensive safety and efficacy profile of ciprofol in mice.

In addition, only intraperitoneal injection is used, and the difference in efficacy between intravenous injection and other commonly used clinical administration routes is not compared. Intravenous injection delivers the drug directly into the bloodstream, with no absorption phase, resulting in rapid achievement of peak plasma concentration, complete bioavailability, and minimal inter-individual variability. In contrast, intraperitoneal injection requires the drug to be absorbed across the peritoneal membrane into the mesenteric circulation, which involves a delayed absorption phase, slower onset, lower and more variable bioavailability, and a prolonged time to peak concentration (Diehl et al., 2001).

Finally, the experimental subjects were only 8-week-old adult mice. The metabolic capacity of young and old mice was different from that of adult individuals, and the appropriate anesthetic dose still needed to be determined separately.

## 5 Conclusion

In the present study, the ED_50_ was 56.014 mg/kg for male and 54.783 mg/kg for female, and the ED_95_ was 91.622 mg/kg for male and 82.212 mg/kg for female. The anesthetic effect of ciprofol in mice exhibited a dose-dependent manner: the higher the dose, the higher the loss of righting reflex rate of anesthesia and the longer the recovery time of anesthesia. Notably, gender had no significant influence on the anesthetic effect of ciprofol in mice. Male and female mice can be anesthetized at the same dose. The ED_50_ and ED_95_ of C57BL/6J mice were determined in this study, which provided a key scientific evidence for the rational dose setting of subsequent ciprofol-related anesthesia experiments in mice, and was of great significance for promoting the standardization and scientification of basic research on ciprofol.

## Data Availability

The raw data supporting the conclusions of this article will be made available by the authors, without undue reservation.
